# Extensive Pollen Flow and Historical Population Expansions Shaped the Phylogeographic Pattern of *Choerospondias axillaris*: Evidence From Chloroplast DNA and ITS Sequences

**DOI:** 10.1002/ece3.73588

**Published:** 2026-04-30

**Authors:** Peng Luo, Jingting Hu, Mengyu Yang, Xuemin Ye, Fei Ding, Jiawei Wang, Nansheng Wu, Rongxi Sun

**Affiliations:** ^1^ School of Forestry Jiangxi Agricultural University Nanchang China; ^2^ Nanchang Urban Ecosystem Research Station Jiangxi Academy of Forestry Nanchang China

**Keywords:** *Choerospondias axillaris*, genetic diversity, phylogeographic pattern, phylogeography, population expansion, suitable habitats

## Abstract

*Choerospondias axillaris* is an ecologically and economically important tree species, yet its phylogeographic pattern remains insufficiently understood. We analysed 29 natural populations of *C. axillaris* in China based on three chloroplast DNA (cpDNA) and internal transcribed spacer (ITS) sequences. The MaxEnt model was then employed to forecast changes in suitable habitats under four climate scenarios. The results show that *C. axillaris* exhibits moderate genetic diversity (cpDNA: *H*_t_ = 0.585; ITS: *H*_t_ = 0.301). CpDNA genetic variation mainly exists among populations (61.51%), whereas ITS genetic variation is primarily concentrated within populations (90.17%). This pattern indicates that seed dispersal is limited, but pollen‐mediated gene flow is extensive. Neutrality tests and mismatch distribution analyses suggest that *C. axillaris* has undergone significant and recent population expansion in its history. We infer that the Xuefeng Mountains‐Wushan region in central China may have served as a significant glacial refuge. After the Last Glacial Maximum, *C. axillaris* expanded southwards (such as Guangdong) from this region to establish the current distribution patterns. Future climate warming may drive its suitable habitats northwards to areas south of the Qinling Mountains. Therefore, priority should be given to protecting identified genetic diversity hotspots and potential refuge populations while implementing targeted management for populations with low genetic variation to ensure the species' evolutionary potential.

## Graphical abstract

MaxEnt‐based predictions reveal dramatic range dynamics of *Choerospondias axillaris* during Quaternary climate cycles, including northward expansion during the last interglacial, southward contraction into fragmented refugia during the last glacial maximum, and subsequent northward recolonization since the post‐glacial period. The predicted glacial refugia spatially coincide with the populations that currently harbour the highest genetic diversity, providing key evidence for their role as long‐term stable refugia. These historical range fluctuations, together with the species' sensitivity to hydrothermal conditions, have jointly shaped its contemporary phylogeographic pattern.

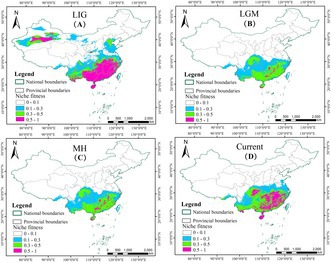

## 1 Introduction

Phylogeography, a discipline emerging from the intersection of evolutionary biology and biogeography, seeks to elucidate the intrinsic relationship between the spatial patterns of species' genetic structure and their historical evolutionary processes (Avise 2000). Since its formal introduction in 1987, phylogeography has shifted biodiversity research from static descriptions based on morphology and fossils towards a comprehensive analysis integrating molecular genetic data with spatiotemporal dynamics (Avise et al. 1987). The core objectives of phylogeography extend beyond examining the geographical distribution patterns of genetic variation among populations (Bobo‐Pinilla et al. 2022), further exploring the timing of species differentiation, migration pathways and the influence of environmental change on biological evolutionary processes (Wei and Zhang 2022). Its research not only focuses on the spatial structure of genetic lineages but also reveals the profound influence of historical geological events, climatic fluctuations and ecological barriers on contemporary biodiversity distribution patterns (RanOmics Group et al. 2024). With the rapid development of molecular marker technologies, the precision of phylogeography research has significantly improved (Amiteye 2021). High‐resolution molecular markers, such as mitochondrial DNA (mtDNA) (Parakatselaki and Ladoukakis 2021), expressed sequence tags‐simple sequence repeats (EST‐SSR) (Jiao et al. 2024) and single nucleotide polymorphisms (SNPs) (Zhang et al. 2023) are widely employed to resolve population genetic structures, infer gene flow and reconstruct historical demographic processes (Lynch and Milligan 1994). In plant phylogeography, chloroplast DNA (cpDNA) represents a particularly valuable marker due to its maternal inheritance, low recombination rate and moderate mutation rate. The geographical distribution of cpDNA haplotypes effectively reflects the historical dispersal processes shaped by seed dispersal (Clegg et al. 1994). By integrating cpDNA haplotype networks with spatial genetic structure information, it is possible to infer the locations of ancient refugia, postglacial migration routes and centres of lineage differentiation (Li et al. 2021).

Concurrently, the emergence of species distribution models (SDMs) has provided a powerful tool for integrating ecological and evolutionary research (Elith and Leathwick 2009). SDMs simulate the spatiotemporal variation of species' potential suitable habitats by establishing statistical relationships between known distribution points and environmental variables. They have become a crucial tool for studying species' responses to climate change and predicting future distribution patterns (Abdulwahab et al. 2022; Rousseau and Betts 2022). Among numerous modelling algorithms, the BIOCLIM model stands as an early representative, pioneering automated species distribution predictions based on climatic factors (Booth et al. 2014; Serrano‐Notivoli et al. 2022). The Maximum Entropy (MaxEnt) model, owing to its stable performance even with limited sample sizes, has become one of the most widely applied modelling approaches today (Warren and Seifert 2011). This model is based on the principle of maximum entropy to select the most uniform probability distribution while satisfying observational constraints, thereby effectively avoiding the overfitting problems of traditional methods when data is sparse (Ahmadi et al. 2023). Moreover, with the deep integration of remote sensing technology and Geographical Information Systems (GIS) (Lü et al. 2019), the environmental variables utilised by SDMs have expanded beyond early climate layers to encompass multidimensional data, including topography, vegetation indices, soil properties and land use. This has significantly enhanced the ecological realism and spatial resolution of the models (Sonwalkar et al. 2010). The widespread sharing of open data has further propelled the synergistic development of phylogeography and species distribution models. Platforms such as the Global Biodiversity Information Facility (GBIF) have collected extensive standardised species distribution records, providing the data foundation for large‐scale distribution modelling (Ivanova and Shashkov 2021). Through multi‐step cleaning and validation of specimen data, researchers are able to achieve relatively high precision ecological niche modelling (Dubos et al. 2022).

At present, interdisciplinary integration is advancing rapidly, enabling multi‐scale analyses from genes to communities by combining molecular genetic data with SDMs (Wang et al. 2014). For example, overlaying the historical suitable habitat distribution maps reconstructed by SDM with areas of genetic diversity hotspots can be used to test hypotheses about species' glacial refugia (Hao et al. 2018). Likewise, combining MaxEnt predictions for future climate scenarios with population genetic models can effectively assess trends in population size changes, local extinction risks and species' adaptive potential to environmental pressures (Zhou et al. 2023). These methods exhibit broad application prospects in endangered species conservation planning, invasive species risk assessment and ecosystem restoration (Collevatti et al. 2012). For example, studies in the mountainous areas of southwestern China have successfully integrated chloroplast DNA, internal transcribed spacer markers and MaxEnt models to identify key areas of high genetic diversity and habitat stability for *Rosa roxburghii* , providing a reliable basis for formulating conservation strategies (He et al. 2025).

*Choerospondias axillaris* (Roxb.) B.L. Burtt & A.W. Hill, a deciduous tree belonging to the Anacardiaceae family, is widely distributed across the subtropical and tropical regions of China and also occurs in India, Japan, Bhutan, Laos, Vietnam and Nepal (Rai et al. 2024; Li et al. 2008). This species is commonly found at elevations between 300 and 2000 m, forming a vital component of subtropical and tropical deciduous broadleaf forest ecosystems. Its fruits are rich in bioactive compounds, including vitamin C, amino acids, flavonoids and polyphenols. As a multipurpose economic tree valued for its fruit production, timber, medicinal properties and ecological restoration properties (Mann et al. 2022). Its holistic utilisation system spans industries such as food processing, biomedical, environmental remediation and ecological construction, demonstrating significant economic returns, sustainable development potential and broad market prospects (Dangal et al. 2023; Zhang et al. 2022). Current research primarily focuses on the chemical composition, pharmacological effects and phenotypic traits of *C. axillaris* (Huang et al. 2025; Li et al. 2022). At the molecular level, preliminary studies employing ISSR markers (Ye et al. 2015), EST‐SSR markers (Xu et al. 2025; Gu et al. 2019) and complete chloroplast genome data (Zhang et al. 2021) have provided essential foundational insights. However, phylogeographic investigations based on chloroplast DNA fragments and nuclear ITS sequences have not yet been conducted.

In this study, we used three chloroplast fragments (*psbA‐trnH, rpl20‐rps12* and *trnL‐trnF*) and an internal transcribed spacer (ITS) sequence, combined with the MaxEnt model, to investigate the genetic evolutionary histories of *C. axillaris* within the complex geological and climatic dynamics of East Asia. The specific research objectives include (1) analysing the genetic diversity levels and population structure characteristics of *C. axillaris* in China; (2) inferring the historical demographic dynamics; (3) determining whether it experienced post‐glacial population expansion or historical bottleneck events; and (4) predicting its potential suitable habitat distribution under different climate scenarios to provide a scientific basis for its resource conservation and sustainable utilisation.

## 2 Materials and Methods

### 2.1 Sampling, DNA Extraction and Sequencing

A total of 254 individual trees of *C. axillaris* were collected from 29 populations spanning 11 provinces and one municipality (Table 1). For each population, fresh young leaves were collected from 4 to 10 sample trees and immediately dried with silica gel. To avoid close relatives, a minimum distance of 50 m was maintained between individuals. Latitude, longitude and altitude of collection sites were recorded using GPS. Genomic DNA was extracted from dried leaves using a modified cetyltrimethyl ammonium bromide (CTAB) method (Li et al. 2013). DNA quality and concentration were assessed by 1% agarose gel electrophoresis and a spectrophotometer (Eppendorf, Hamburg, Germany), respectively.

**TABLE 1:** Sampling information for 29 populations of *Choerospondias axillaris* .

Population	Location	Number	Longitude (E)	Latitude (N)	Altitude (m)
JXYF	Yifeng, Jiangxi, China	10	114.79	28.39	261
JXCY	Chongyi, Jiangxi, China	10	114.31	25.68	459
JXLN	Longnan, Jiangxi, China	9	114.79	24.91	444
JXGF	Guangfeng, Jiangxi, China	10	118.18	28.43	364
JXXY	Xinyu, Jiangxi, China	8	114.91	27.82	589
JXYG	Yugan, Jiangxi, China	10	116.68	28.71	394
HNNX	Ningxiang, Hunan, China	10	112.54	28.27	71
HNAH	Anhua, Hunan, China	10	111.21	28.37	408
HNTY	Taoyuan, Hunan, China	10	111.48	28.91	229
HNYS	Yongshun, Hunan, China	10	109.85	28.98	1437
HNXT	Xiangtan, Hunan, China	7	112.93	27.83	68
HBZG	Zigui, Hubei, China	6	110.97	30.82	602
HBXA	Xian'an, Hubei, China	10	114.29	29.85	127
HBCB	Chibi, Hubei, China	8	113.62	29.87	67
HBCY	Chongyang, Hubei, China	8	114.03	29.55	696
HBTS	Tongshan, Hubei, China	8	114.47	29.61	804
GXSL	Shanglin, Guangxi, China	4	108.61	23.43	271
GXYS	Yangshuo, Guangxi, China	6	110.49	24.78	356
GXZS	Zhongshan, Guangxi, China	9	111.29	24.52	1785
GXCW	Cangwu, Guangxi, China	8	111.54	23.86	168
GXPN	Pingnan, Guangxi, China	10	110.38	23.54	1374
HNQZ	Qiongzhong, Hainan, China	10	109.83	19.03	569
GDLC	Lechang, Guangdong, China	7	113.34	25.13	104
FJMX	Mingxi, Fujian, China	7	117.19	26.35	517
ZJKH	Kaihua, Zhejiang, China	10	118.41	29.13	378
AHQM	Qimen, Anhui, China	10	117.71	29.85	152
YNMS	Mangshi, Yunnan, China	10	98.59	24.44	933
GZJK	Jiangkou, Guizhou, China	9	108.83	27.71	1576
CQSPB	Shapingba, Chongqing, China	10	106.45	29.54	334

After screening variation in thirty cpDNA gene fragments and 10 nuclear gene fragments, three cpDNA gene fragments: *psbA‐trnH* (Sang et al. 1997), *rpl20‐rps12* (Shaw et al. 2007) and *trnL‐trnF* (Taberlet 1991), as well as one pair of nuclear gene fragments, *ITS* (White et al. 1990), were selected for amplification and sequencing (Table S1). Polymerase chain reaction (PCR) amplification was conducted using a 25 μL reaction mixture comprising 12.5 μL of 2× Taq PCR Master Mix, 1 μL each of forward and reverse primers, 1 μL of template DNA and 9.5 μL of ddH₂O. PCR amplification followed a ‘low‐temperature slow‐ramping’ protocol (Shaw et al. 2007) with the following steps: initial denaturation at 95°C for 5 min; 30 cycles of denaturation at 95°C for 1 min, annealing at 50°C for 1 min and extension at 65°C for 4 min; final extension at 65°C for 5 min; and product storage at 4°C. The PCR products were purified and sequenced by Hunan QingKe Biotechnology Co. Ltd.

All PCR products were subjected to direct sequencing. Chromatogram analysis revealed no significant multiple peaks, indicating relative sequence homogeneity within individuals. Although direct sequencing may fail to detect weak variations, the haplotype data obtained remain suitable for population‐level phylogeographic analyses due to this study's focus on inter‐population variation patterns and the consistent processing methods applied to all samples. The length of the conserved ITS sequence used for analysis is 270 base pairs. Following BLASTn alignment, this fragment corresponds to a portion of the ITS region within the complete ITS sequence of *Harpephyllum caffrum* (GenBank accession number: KF664197.1), encompassing a partial segment of ITS1 (227 bp) and the core region at the 5′ end of the 5.8S (43 bp). The selection of this conserved region ensures the reliability of alignment across all samples.

### 2.2 Population Genetics Analysis

All ABI format sequencing chromatograms were manually checked using BioEdit 7.0.9 (Hall 1999). Import the edited sequences into MEGA 11.0.13 (Tamura et al. 2013) and perform multiple sequence alignment using the Align by ClustalW module, with the default settings (Gap Opening Penalty = 15.00, Gap Extension Penalty = 6.66). Trim the less conserved regions at both ends of the sequence. Finally, the three processed cpDNA fragments were assembled using PhyloSuite 1.2.3 (Zhang et al. 2020) to generate a complete haplotype sequence for subsequent analyses.

Population genetic diversity indices, including haplotype diversity (*H*_d_) and nucleotide diversity (*π*), were calculated using DnaSP 5.0 (Rozas et al. 2003). Use the software's ‘define sequence sets’ function to group all sequences to identify different haplotypes, and export the haplotype lists and polymorphic site information. To test the neutral evolution hypothesis, Tajima's *D* (Tajima 1997) and Fu's Fs statistics (Fu 1997) were calculated using DnaSP 5.0 Furthermore, based on the ‘population spatial expansion model’, mismatch distribution curves were plotted using DnaSP 5.0 and the fit of the observed distribution to the expected distribution was assessed through 1000 bootstrap resamples to infer historical population expansion events.

Analysis Molecular variance (AMOVA) was conducted using Arlequin 3.11.0 (Meirmans 2012; Excoffier and Lischer 2010). In the analysis settings, all 29 populations were defined as a single cluster. Based on the pairwise difference matrix, the fixation index (*F*_st_) of population genetic differentiation was calculated with 1000 permutations. Gene flow (*N*_m_) was estimated using the formula *N*_m_ = (1 − *F*_st_)/4*F*_st_. Concurrently, the software was used to calculate the sum of squared deviations (*SSD*) and Harpending's raggedness index (*Hrag*) of mismatch distribution (Harpending 1994), with significance assessed through 1000 bootstrap tests. The system's geographical structure was assessed using PermutCpSSR 2.0 (Pons and Petit 1996). Haplotype sequences and frequency data were input, and following 1000 permutation tests, total genetic diversity (*H*_t_), within‐population genetic diversity (*H*_s_) and genetic differentiation indices (*N*_st_, *G*_st_) were calculated.

Using POPART 1.7.0 (Leigh and Bryant 2015) and the statistical simplification network method (TCS) (Clement et al. 2002), haplotype networks were constructed with a 95% simplification connection threshold. ArcGIS 10.8.1 (Guo et al. 2014) was used to visualise haplotype frequencies across different populations, generating haplotype geographical distribution maps. To test the isolation by distance (IBD) effect, pairwise genetic distances among all populations were first calculated using the Kimura 2‐parameter (K2P) (Kimura 1980) model in MEGA 11.0.13, and a genetic distance matrix was generated. Subsequently, in GenAlEx 6.5 (Peakall and Smouse 2010), a geographic distance matrix was calculated based on the latitude and longitude coordinates of sampling points. This matrix underwent logarithmic transformation [Log(1 + geographic distance)] to reduce bias caused by outliers. Finally, a Mantel test was conducted in GenAlEx 6.5 (José Alexandre et al. 2013) to assess the correlation between the pairwise genetic distance (*F*_st_/(1‐*F*_st_)) matrix and the geographic distance (ln‐transformed) matrix, with 1000 permutations performed to evaluate statistical significance.

### 2.3 Species Distribution Modelling

Based on the species distribution points recorded in this study and data from the Chinese Virtual Herbarium (https://www.cvh.ac.cn/) (Table S2), to avoid data duplication, mitigate geographic collection biases and reduce model overfitting caused by spatial autocorrelation, we implemented a rigorous data cleaning protocol: (1) All records were manually verified to ensure taxonomic accuracy and coordinate reliability; (2) Obvious climatic outliers were removed; (3) Spatially overlapping data points within 10 km were eliminated (Boria et al. 2014). A total of 144 valid occurrence records of *C. axillaris* were obtained, which better covered the distribution range of *C. axillaris* in China and were suitable for model building. A total of 19 bioclimate variables with a spatial resolution of 2.5 arcminutes were downloaded from the WorldClim database (http://www.worldclim.org/) (Table S3), covering four periods: the Last Interglacial (LIG; 120–140 ka BP), the Last Glacial Maximum (LGM; 22 ka BP), the Mid‐Holocene (MH; 6 ka BP) and the Current (1970–2000). The MaxEnt 3.4.4 model was used to reconstruct the potential geographical distribution of *C. axillaris* during the LIG, LGM, MH and the Current (Phillips and Miroslav 2008). This multi‐step data cleaning and spatial filtering process, prior to model calibration, is a critical step to minimise model overfitting and enhance model reliability. To avoid overfitting and multicollinearity, Spearman's rank correlation analysis was used to see how two variables were connected. One variable was removed if the absolute correlation coefficient (|*r*|) between two variables was ≥ 0.8 (Jin et al. 2008). Preliminarily screened environmental variable layer files were processed into ASCII format using ArcGIS 10.8.1 and imported into the Maxent 3.4.4 model for simulation. This process was repeated 10 times using the standard settings. The jackknife test was then used to remove variables that were not important. Ten environmental variables were ultimately selected as climate predictors for ecological niche modelling. We evaluated model performance using the area under the receiver operating characteristic curve (AUC). An AUC range of 0.6–0.7 indicates poor performance; a range of 0.7–0.8 indicates average performance; a range of 0.8–0.9 indicates good performance; and a range of 0.9–1 indicates excellent performance (Lobo et al. 2008). To assess the predictive performance of the model, 25% of the distribution points were randomly selected as the test set and the remaining 75% as the training set. The model was run with 10 repetitions and 500 iterations per run, using a convergence threshold of 0.00001, with all other parameters kept at their default settings. The prediction results were imported into ArcGIS 10.8.1 for suitability classification, where habitat suitability was categorised into four classes based on the habitat suitability index: unsuitable (0–0.1), low suitability (0.1–0.3), moderately suitable (0.3–0.5) and highly suitable (0.5–1) (Liu et al. 2025). This fixed classification scheme was employed to visualise the continuous gradient of habitat suitability and to ensure consistent criteria for comparing habitat quality across different historical periods. Finally, statistical analyses were performed to quantify the potential distribution areas under four main climate scenarios across different time periods.

## 3 Results

### 3.1 Haplotype Distribution

**FIGURE 1:**
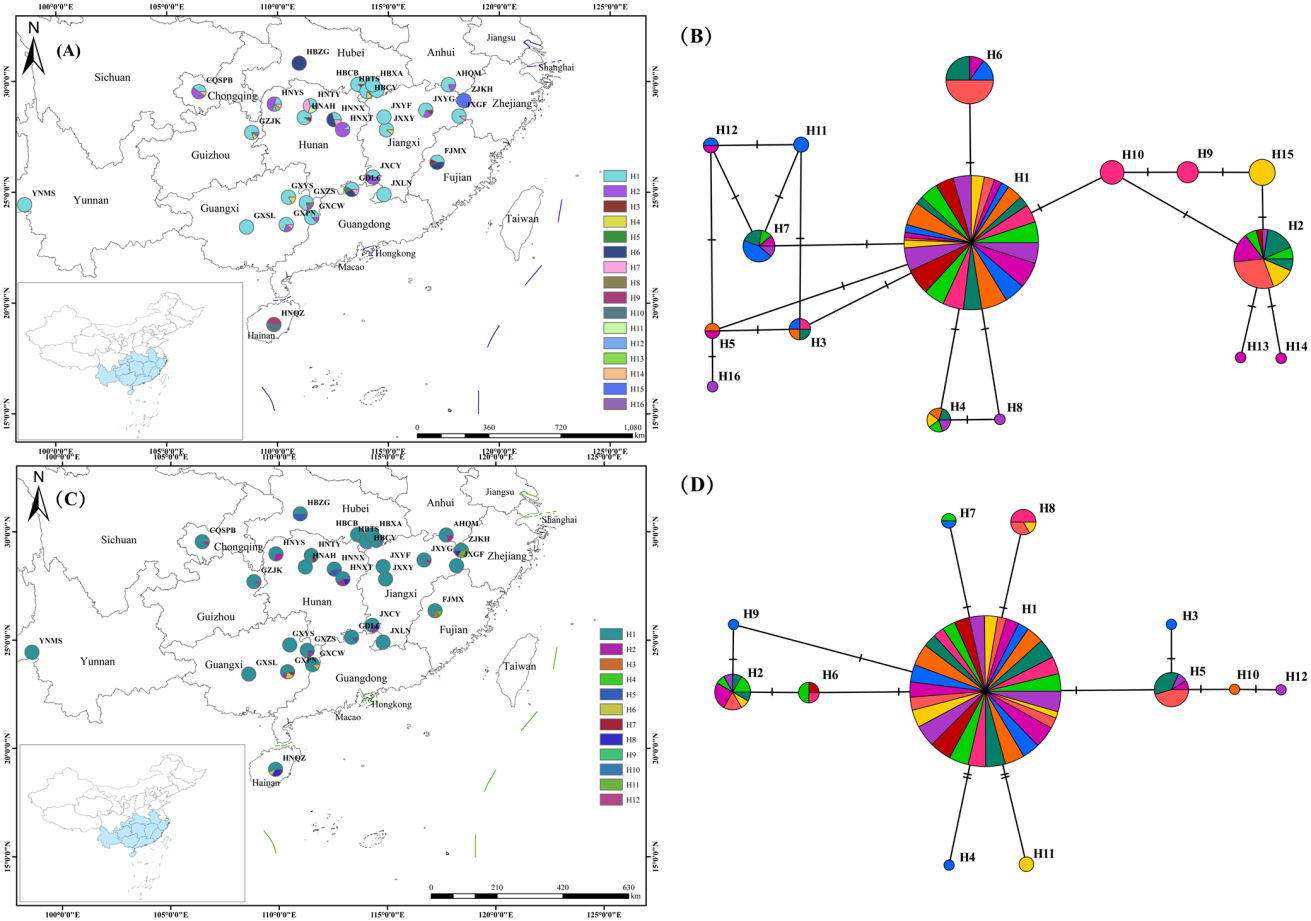
Geographic distribution map of haplotypes and haplotype network diagram of *Choerospondias axillaris* . (A) Geographic distribution map of haplotypes based on cpDNA. (B) Haplotype network diagram based on cpDNA. (C) Geographic distribution map of haplotypes based on ITS. (D) Haplotype network diagram based on ITS. Different colours in the (B) and (D) distinguish different populations; the haplotype frequency is indicated by the size of the circle.

The total aligned sequence of three cpDNA fragments was 2210 bp. Based on the concatenated sequences, 12 variation sites were detected, including eight nucleotide substitutions and four indels (Table S4). A total of 16 cpDNA haplotypes (H1‐H16) were detected from 29 populations (Figure 1A). 21 of these populations exhibited more than one haplotype, including one population (HNTY) with five haplotypes, two populations (GDLC and HNAH) with four haplotypes each, eight populations (CQSPB, HBCY, FJMX, GXZS, GXPN, HNQZ, JXYF and ZJKH) with three haplotypes each, and 10 populations (AHQM, HBCB, GXYS, GXCW, GZJK, HNNX, HNYS, HNXT, JXCY and JXLN) with two haplotypes each, while the remaining eight populations (GXSL, JXGF, JXXY, JXYG, HBTS, HBXA, YNMS and HBZG) were fixed for one haplotype. Notably, eight unique haplotypes were identified in the six populations (GXZS, GZJK, HNAH, HNTY, HBZG and ZJKH) (Table 2). The GDLC population exhibited the highest haplotype diversity. CpDNA haplotype H1 was the most prevalent distributed haplotype, detected in 63% (160 individuals) of the individuals occurring in 26 populations, which was presumed to be a main haplotype because of its high dispersal capacity and location at the interior nodes of the haplotype network. The second (H2) and third (H6) most frequent haplotypes were detected in 12% (31 individuals) and 8% (20 individuals) of the individuals, respectively (Figure 1B). The aligned ITS sequence was 270 bp, containing 12 variable sites (Table S5). A total of 12 ITS haplotypes (H1‐H12) were identified (Figure 1C). The GXPN population harboured four haplotypes, seven populations (FJMX, GXZS, GZJK, HNAH, HNYS, HNXT and HBZG) with three haplotypes each, and 10 populations (AHQM, CQSPB, GDLC, GXCW, HNQZ, HNTY, JXYF, JXYG, YNMS and ZJKH) with two haplotypes each, while the remaining 11 populations (HBCB, HBCY, GXSL, GXYS, HNNX, JXCY, JXGF, JXLN, JXXY, HBTS and HBXA) were fixed for one haplotype. Five unique haplotypes were detected in five populations (FJMX, HNAH, JXYG, HBZG and ZJKH). The HBZG population displayed the highest haplotype diversity. ITS haplotype H1 was the most frequent haplotype, appearing in 83% (211 individuals) of individuals distributed in all 29 populations. H1 was a main haplotype because it was scattered in all populations and located at a central position in the network (Figure 1D).

**TABLE 2:** cpDNA and ITS haplotype and genetic diversity in 29 populations of *Choerospondias axillaris* .

Population	cpDNA			ITS		
	Haplotype (number)	*H*_d_	*π*	Haplotype (number)	*H*_d_	*π*
AHQM	H1(8)H2(2)	0.35556	0.00033	H1(8)H2(2)	0.35556	0.00263
HBCB	H1(7)H3(1)	0.25000	0.00012	H1(8)	0.00000	0.00000
CQSPB	H1(4)H2(5)H4(1)	0.64444	0.00061	H1(9)H2(1)	0.20000	0.00148
HBCY	H1(6)H4(1)H5(1)	0.46429	0.00023	H1(8)	0.00000	0.00000
FJMX	H1(3)H3(1)H6(3)	0.71429	0.00040	H1(5)**H3(1)H4(1)**	0.52381	0.00423
GDLC	H1(3)H5(1)H6(2)H7(1)	0.80952	0.00049	H1(6)H5(1)	0.28571	0.00106
GXSL	H1(4)	0.00000	0.00000	H1(4)	0.00000	0.00000
GXYS	H1(5)H4(1)	0.33333	0.00015	H1(6)	0.00000	0.00000
GXZS	H1(7)H2(1)**H8(1)**	0.41667	0.00031	H1(7)H2(1)H5(1)	0.41667	0.00247
GXCW	H1(7)H2(1)	0.25000	0.00023	H1(7)H6(1)	0.25000	0.00093
GXPN	H1(7)H2(2)H7(1)	0.51111	0.00042	H1(6)H2(1)H6(2)H7(1)	0.64444	0.00321
GZJK	**H9(4)H10(5)**	0.55556	0.00026	H1(5)H6(1)H8(3)	0.63889	0.00267
HNQZ	H1(3)H6(5)H7(2)	0.68889	0.00042	H1(6)H5(4)	0.53333	0.00198
HNNX	H1(9)H3(1)	0.20000	0.00009	H1(10)	0.00000	0.00000
HNAH	H1(3)H7(4)**H11(2)**H12(1)	0.77778	0.00045	H1(8)H7(1)**H9(1)**	0.37778	0.00148
HNTY	H1(2)H2(5)H12(1)**H13(1)H14(1)**	0.75556	0.00078	H1(7)H2(3)	0.46667	0.00346
HNYS	H1(1)H2(9)	0.20000	0.00019	H1(6)H2(2)H8(2)	0.62222	0.00395
HNXT	H1(3)H2(4)	0.57143	0.00053	H1(5)H2(1)H8(1)	0.52381	0.00317
JXCY	H1(9)H7(1)	0.20000	0.00009	H1(10)	0.00000	0.00000
JXGF	H1(10)	0.00000	0.00000	H1(10)	0.00000	0.00000
JXLN	H1(8)H4(1)	0.22222	0.00010	H1(9)	0.00000	0.00000
JXXY	H1(8)	0.00000	0.00000	H1(8)	0.00000	0.00000
JXYF	H1(7)H2(2)H3(1)	0.51111	0.00042	H1(9)H2(1)	0.20000	0.00148
JXYG	H1(10)	0.00000	0.00000	H1(9)**H10(1)**	0.20000	0.00148
HBTS	H1(8)	0.00000	0.00000	H1(8)	0.00000	0.00000
HBXA	H1(10)	0.00000	0.00000	H1(10)	0.00000	0.00000
YNMS	H6(10)	0.00000	0.00000	H1(5)H5(5)	0.55556	0.00206
HBZG	**H15(6)**	0.00000	0.00000	H1(3)H8(1)**H11(2)**	0.73333	0.00519
ZJKH	H1(8)H4(1)**H16(1)**	0.37778	0.00028	H1(9)**H12(1)**	0.20000	0.00222
Total	—	0.58106	0.00048	—	0.30581	0.00174

*Note:* () in bold indicates unique haplotype.

### 3.2 Population Genetic Diversity

The cpDNA exhibited moderate genetic diversity (*H*_d_: 0.58106, *π*: 0.00048, *H*_t_: 0.585, *H*_s_: 0.338), with the GDLC, HNAH, HNTY and FJMX populations possessing significantly higher haplotype diversity than the remaining populations. For ITS, the genetic diversity indices (*H*_d_: 0.30581, *π*: 0.00174, *H*_t_: 0.301, *H*_s_: 0.266), as well as the HBZG, GXPN, GZJK and HNYS populations displayed notably higher haplotype diversity compared to other populations. At the species level, the overall genetic diversity parameters (*H*_d_, *H*_t_, *H*_s_) for cpDNA were higher than those for ITS, while the total nucleotide diversity (*π*) of ITS was higher than that of cpDNA (Table 2).

### 3.3 Population Genetic Differentiation and Genetic Structure

Analysis of molecular variance (AMOVA) of cpDNA revealed that the percentage of genetic variation among populations (61.51%) was substantially greater than that within populations (38.49%). The genetic differentiation coefficient (*F*_st_: 0.6151, *p* < 0.001) indicated that most chloroplast genetic variation in *C. axillaris* occurred among the populations (Table 3). The permutation test further showed that the overall genetic differentiation coefficient (*N*_st_: 0.518) was significantly higher than the inter‐population genetic differentiation coefficient (*G*_st_: 0.422), indicating a significant phylogeographic structure in *C. axillaris* . The gene flow estimate (*N*_m_: 0.16) suggested highly restricted genetic exchange among populations, thereby promoting genetic divergence.

**TABLE 3:** Analysis of Molecular Variance (AMOVA) for cpDNA and ITS of *Choerospondias axillaris* .

Source of variation	df	Sum of squares	Variance components	Percentage of variation	Differentiation coefficient
cpDNA					
Among populations	28	947.487	3.60981 Va	61.51	0.6151
Within populations	225	508.285	2.25904 Vb	38.49	
Total	253	1455.772	5.86886		
ITS					
Among populations	28	11.596	0.02311 Va	9.83	0.0983
Within populations	225	47.688	0.21195 Vb	90.17	
Total	253	59.283	0.23506		

In contrast, AMOVA results based on ITS showed that the genetic variation among populations (9.83%) was substantially lower than that within populations (90.17%). The genetic differentiation coefficient (*F*_st_: 0.0983, *p* < 0.001) was extremely low, suggesting weak genetic differentiation among populations and that most ITS genetic variation was maintained within populations (Table 3). Additionally, *N*_st_ (0.097) was lower than *G*_st_ (0.116), suggesting the absence of a significant phylogeographic structure for ITS. The gene flow estimate (*N*_m_: 2.29) suggests frequent genetic exchange among populations, which inhibits genetic differentiation.

**FIGURE 2:**
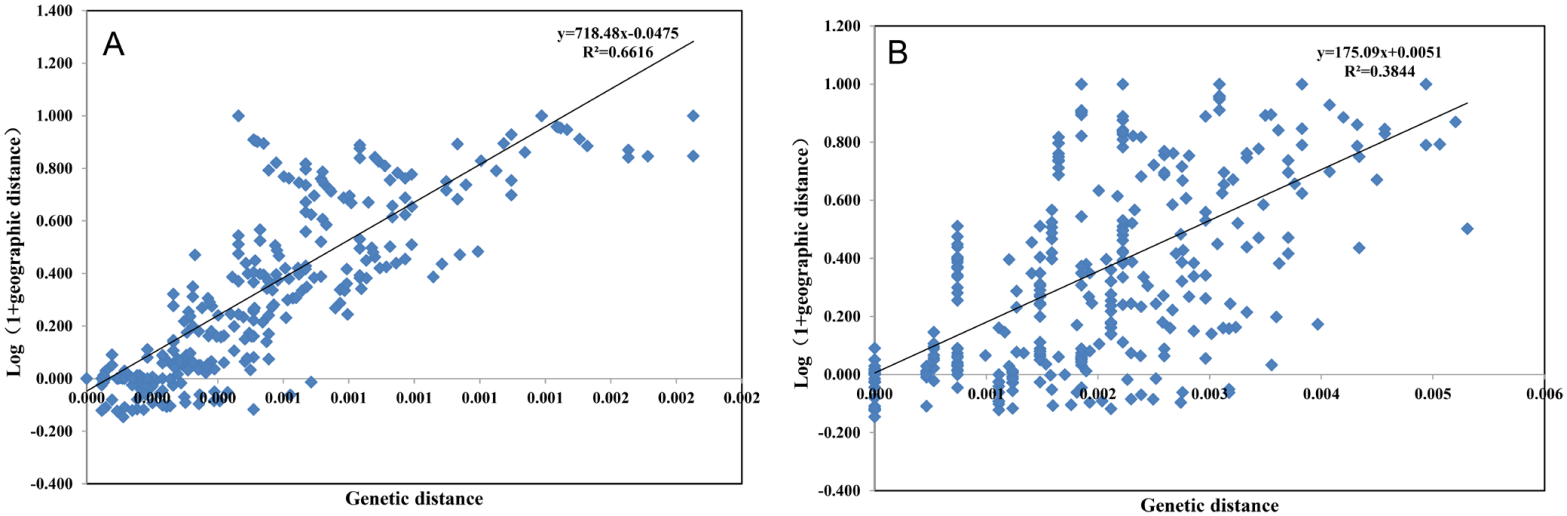
Mantel test of Log(1 + geographic distance) and genetic distance among populations of *Choerospondias axillaris* based on cpDNA (A) and ITS (B). The Mantel test correlation was not significant for cpDNA (*r*: −0.256, *p* = 1.000) or ITS (*r*: −0.254, *p* = 1.000).

Mantel analysis revealed that both cpDNA (*r*: −0.256, *p* = 1.000) and ITS (*r*: −0.254, *p* = 1.000) exhibited weak and statistically non‐significant correlations between Log(1 + geographic distance) and genetic distance. Although linear regression analyses yielded relatively high coefficients of determination (cpDNA:*R* ^2^ = 0.6616; ITS:*R* ^2^ = 0.3814, Figure 2), the paired distance data points used in the regression are not statistically independent; this violates the fundamental assumptions of regression analysis. Therefore, the significance and the R^2^ values from the regression are potentially unreliable and likely overestimated. Consequently, we rely on the Mantel test results (*p* = 1.000) as the primary statistical evidence, which indicates no significant relationship between genetic and geographic distance. Thus, we conclude that geographic distance is not the main factor driving the genetic differentiation of *C. axillaris* populations.

### 3.4 Population Historical Dynamics

**FIGURE 3:**
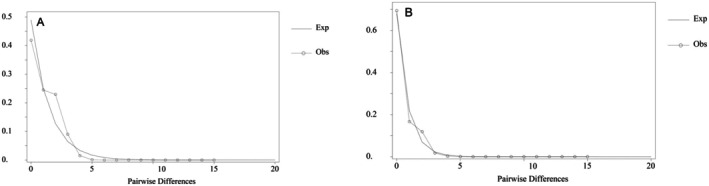
Mismatch distribution diagram of *Choerospondias axillaris* based on cpDNA (A) and ITS (B). The observed mismatch distribution did not differ significantly from the expectation under the spatial expansion model for either cpDNA (*SSD*: 0.02472, *p* = 0.174; *Hrag*: 0.25238, *p* = 0.477) or ITS (*SSD*: 0.02071, *p* = 0.161; *Hrag*: 0.24672, *p* = 0.412).

For cpDNA data, although Tajima's D value (−0.82506, *p* > 0.10) was not significant, Fu's Fs statistic (−8.856, *p* < 0.001) was significantly negative. Given that Fu's Fs are particularly sensitive to signals of population expansion, this result strongly suggests that the population experienced a rapid and recent expansion. For ITS data, both Tajima's D value and Fu's Fs statistic were significantly negative (Tajima's *D*: −1.80305, *p* < 0.05; Fu's Fs: −10.320, *p* < 0.001), providing strong and dual evidence for recent population expansion. Mismatch distribution analyses further confirmed the above conclusions. Both molecular markers exhibited single peak distributions, with observed values highly consistent with those predicted by the spatial expansion model (Figure 3). This intuitive result is quantitatively supported by goodness‐of‐fit tests: cpDNA (*SSD*: 0.02472, *p* = 0.174 > 0.05; *Hrag*: 0.25238, *p* = 0.477 > 0.05) and ITS (*SSD*: 0.02071, *p* = 0.161 > 0.05; *Hrag*: 0.24672, *p* = 0.412 > 0.05). Both *SSD* and *Hrag* values were not significant. This indicates that there is no significant deviation between the observed distribution and the predictions of the expansion model, thereby supporting the hypothesis of population expansion. In summary, the significant negative values in neutral tests (particularly Fu's Fs), combined with the unimodal shape of the mismatch distribution and the model's favourable fit, all point to a clear conclusion: the *C. axillaris* population has indeed undergone a rapid expansion event in its history.

### 3.5 Changes in Species Distribution

**FIGURE 4:**
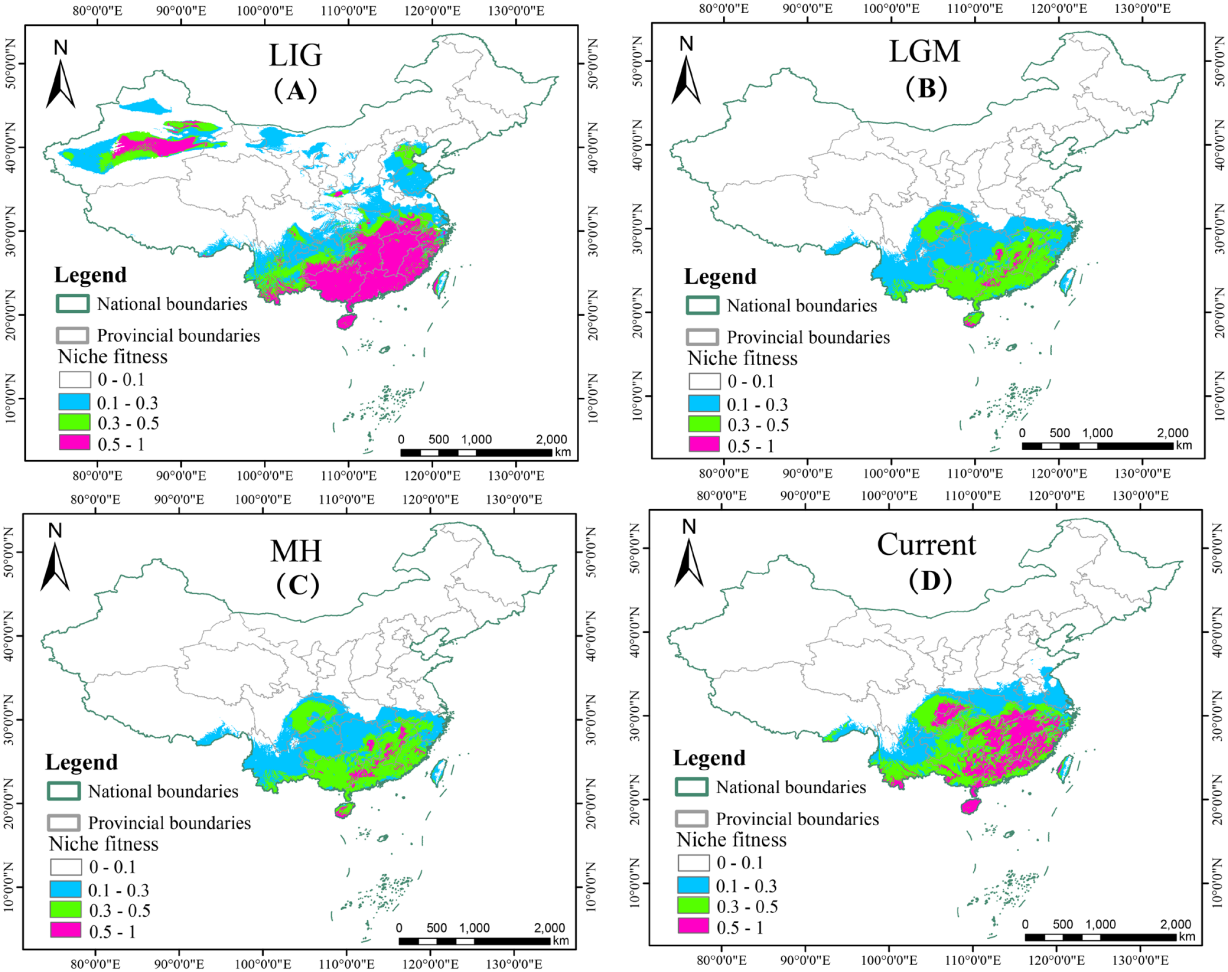
Potential suitable habitats of *Choerospondias axillaris* at different times. (A) Last Interglacial (LIG). (B) Last Glacial Maximum (LGM). (C) Mid‐Holocene (MH). (D) Current. Colours represent habitat suitability: White (unsuitable, 0–0.1), blue (low suitability, 0.1–0.3), green (moderately suitable, 0.3–0.5) and pink (highly suitable, 0.5–1).

Spearman's correlation analysis (Figure S1) selected ten environmental variables for modelling *C. axillaris* distribution: Bio2, Bio3, Bio5, Bio6, Bio7, Bio8, Bio12, Bio14, Bio15 and Bio18 (Figure S2). The area under the curve (AUC) value was 0.929, indicating high predictive accuracy (Figure S3). The five most influential environmental variables across four periods were annual precipitation (Bio12), precipitation of the driest month (Bio14), maximum temperature of the warmest month (Bio5), minimum temperature of the coldest month (Bio6) and precipitation of the warmest quarter (Bio18) (Table S6). According to the environmental variable response curves, annual precipitation and precipitation of the driest month are key factors dominating the distribution of *C. axillaris* . During years with higher precipitation, the probability of *C. axillaris* occurrence increased accordingly, indicating its suitability for growth in humid environments. When the driest month's precipitation is insufficient, the probability of *C. axillaris* occurrence decreased significantly, reflecting this species' clear preference for habitats with a stable water supply and no distinct dry season (Figure S4). The results indicated that the distribution of *C. axillaris* was strongly influenced by precipitation and temperature. During the LIG (Figure 4A), the total potential suitable area for *C. axillaris* reached its maximum range, with highly suitable habitats concentrated in northwestern and southeastern China. In contrast, during the LGM (Figure 4B), pronounced global cooling caused a marked contraction of suitable area compared with the LIG and many previously highly suitable regions became unsuitable. In the MH (Figure 4C), increasing warmth and humidity facilitated the expansion of highly suitable regions relative to the LGM. Under current climatic conditions (Figure 4D), the potential distribution of *C. axillaris* is mainly located south of the Qinling‐Huaihe Line in China, closely corresponding to its actual distribution. Compared with the MH, the extent of highly suitable area has continued to increase and demonstrates a northward expansion trend (Table S7). Overall, the model results indicate contraction‐expansion dynamics through time. Annual precipitation was the most influential variable, indicating that precipitation plays a key role in shaping species distributions and is linked to the reproductive cycle of many plants (Chagas et al. 2020; Sales et al. 2025).

## 4 Discussion

### 4.1 Genetic Diversity of *C. axillaris*

Analysis based on chloroplast gene fragments indicates that *C. axillaris* exhibits moderate genetic diversity at the species level. The overall haplotype diversity (*H*_d_: 0.581), the total nucleotide diversity (*π*: 0.00048) and the total genetic diversity (*H*_t_: 0.585) which are lower than the average (*H*_t_: 0.670) reported across 170 species analysed using chloroplast DNA markers (Petit et al. 2005). Unpublished SRR data (*H*_e_: 0.582; *I*: 1.139) indicating moderate genetic variation further corroborates the aforementioned findings. The cpDNA haplotype diversity of *C. axillaris* was higher than that of *Verbena officinalis* (*H*_d_: 0.515; *π*: 0.00116) (Ye 2023), *Prunus serrulata* (*H*_d_: 0.553; *π*: 0.00136) (Peng 2021) and *Toxicodendron vernicifluum* (*H*_d_: 0.301; *π*: 0.00087) (Wang 2022). Among the 29 populations studied, the GDLC, HNAH and HNTY populations exhibited higher haplotype diversity. Concurrently, Maxent modelling indicated that these regions were highly suitable habitats for *C. axillaris* during glacial periods, suggesting they may have been genetic diversity hotspots or potential glacial refugia for *C. axillaris* . Conversely, eight populations exhibited no detectable genetic variation, likely attributable to founder effects, which would diminish their evolutionary potential and adaptability (Uller and Leimu 2011; Barrett and Charlesworth 1991). Our results indicate that *C. axillaris* possesses moderate levels of cpDNA diversity, which may be attributable to the fact that cpDNA is maternally inherited and dispersed primarily through seeds (Walbot and Evans 2003; Cain et al. 2000). The fruits of *C. axillaris* easily fall off when mature and can be dispersed through water or animals, facilitating gene flow among populations to some extent. Moreover, the chloroplast genome is highly conserved and evolves slowly, with a low mutation rate, thereby limiting the accumulation of chloroplast genetic diversity (Daniell et al. 2016; Howe et al. 2003).

In contrast, analysis based on nuclear ITS sequences revealed that although the absolute diversity index at the species level (*H*t = 0.301) is lower than that of cpDNA, its value remains higher than the average for 77 angiosperm nuclear gene markers (*H*_t_: 0.137) (Petit et al. 2005), indicating the presence of relatively high nuclear ITS genetic diversity. The ITS diversity of *C. axillaris* was higher than that of *Chamaesium paradoxum* (*H*_d_: 0.255; *π*: 0.00100) (Zheng 2021a). Among these populations, the HBZG population exhibited the highest haplotype diversity, indicating that it possesses rich genetic resources and strong evolutionary adaptive potential. The GXPN and GZJK populations showed the next highest diversity levels, which may be attributable to relatively stable local environments or active gene flow. However, no haplotype or nucleotide variation was detected in ITS among the 11 populations, highlighting the need for targeted conservation measures to prevent genetic homogenisation and the associated loss of adaptive potential (Salgotra and Chauhan 2023; Rao 2004). The HNQZ population exhibited relatively high genetic diversity in both cpDNA and ITS analyses. Given its geographic isolation on Hainan Island, this population may have achieved greater long‐term stability.

Notably, cpDNA data indicated that southeastern populations (such as GDLC) exhibit the highest genetic diversity, whereas ITS data showed that central populations (such as HBZG) possess the greatest genetic diversity. This inconsistency may reflect regionally specific adaptive differentiation or the differential effects of selective pressures acting on chloroplast versus nuclear markers (Kimura 1968; Lynch et al. 2016). These differences are also closely linked to the biological characteristics of *C. axillaris* . As a deciduous tree widely distributed across southern China, its strong ecological adaptability, preference for moist habitats and relatively large population size contribute to the maintenance of high levels of genetic variation (Li et al. 2008). In addition, nuclear genes are biparentally inherited and subject to genetic recombination through mechanisms such as linkage exchange and independent assortment. This process continually generates new allelic combinations and increases the genetic variation of populations (Hare 2001; Clegg et al. 1997). *C. axillaris* is dioecious and strictly outcrossing, with pollen dispersed primarily by insects such as flies and bees, sometimes assisted by water (Yang et al. 2023; Dangal et al. 2023). This frequent cross‐pollination effectively maintains and increases genetic diversity, thereby enhancing the adaptive potential of the population.

### 4.2 Genetic Structure and Genetic Differentiation of *C. axillaris*

Research based on cpDNA fragments revealed that the genetic differentiation coefficient *N*_st_ (0.650) was significantly higher than *G*_st_ (0.422). This indicates a pronounced phylogeographic structure among populations. This pattern is consistent with *Quercus franchetii* (*H*_t_: 0.982, *H*_s_: 0.123; *N*_st_: 0.959 > *G*_st_: 0.874) (Zheng 2021b), *Periploca sepium* (*H*_*t*_: 0.933, *H*_s_: 0.410; *N*_st_: 0.585 > *G*_st_: 0.561) (Yang 2022) and *Chimonobambusa utilis* (*H*_t_: 0.956, *H*_s_: 0.507; *N*_st_: 0.976 > *G*_st_: 0.470) (Liu 2022). AMOVA results confirmed that cpDNA genetic variation mainly exists among populations (*F*_st_ = 0.6151, *p* < 0.01). This conclusion is supported by the observation that *H*_t_ (0.585) was substantially higher than *H*_s_ (0.338). Although the *G*_st_ value for *C. axillaris* was lower than the mean chloroplast *G*_st_ value (0.637) of 124 angiosperms reported by Petit et al. (2005), the relatively high *F*_st_ (0.6151) and extremely low estimated gene flow (*N*_m_: 0.16) still indicate a high level of genetic differentiation among populations. This relatively low level of differentiation compared to the average in angiosperms may be related to the effective dispersal mechanisms of its seeds. Although the sampling range spans from Yunnan to Zhejiang, geographical distance may limit seed‐mediated gene flow; however, the inherent dispersibility of seeds may partially offset the effects of geographical isolation. The observed phylogeographic structure likely reflects the residual effects of historical population establishment or fragmentation (Ehrlén 2000; Ferris et al. 1999). The extremely low gene flow (*N*_m_: 0.16) can amplify the effects of genetic drift, promoting local adaptation and population differentiation and ultimately lead to a highly structured genetic pattern (Spieth 1974; Wainberg et al. 2024).

For ITS, the *G*_st_ (0.116) was lower than the average *G*_st_ (0.150) summarised by Petit for 22 plant species based on parental genetic markers (Petit et al. 2005). *N*_st_ (0.097) was slightly lower than *G*_st_, indicating an absence of pronounced phylogeographic structure among populations. AMOVA results further demonstrated that genetic variation was predominantly distributed within populations (90.17%), with only 9.83% occurring among populations. This finding is consistent with AMOVA analyses results of *Parthenocissus tricuspidata* (Wu 2022a) and *Vitex rotundifolia* (Wu 2022b). The extremely low genetic differentiation coefficient (*F*_st_ = 0.0983, *p* < 0.001) combined with high gene flow (*N*_m_: 2.29) indicates frequent genetic exchange among populations. This extensive gene flow is mainly attributable to the species' seed dispersal via water or animals, as well as substantial pollen‐mediated gene movement (Sexton et al. 2014; Culley et al. 2002). The extensive flow of pollen may have entirely smoothed out any weak signals of differentiation arising from geographical distance, leading to genetic variation being concentrated primarily within populations. Therefore, despite the extensive geographical distribution of *C. axillaris* , no obvious phylogeographic structure was detected based on ITS sequences. It should be noted that ITS, as a multicopy nuclear gene marker, may be subject to incomplete lineage sorting, which could potentially account for the discrepancies observed between its genetic structure and that revealed by cpDNA.

AMOVA results based on ITS data indicate that genetic variation in *C. axillaris* mainly exists within populations, whereas results based on cpDNA data yield the opposite conclusion. This indicates that the current spatial genetic structure of *C. axillaris* is jointly shaped by extensive pollen‐mediated gene flow and limited seed‐mediated gene flow, which is similar to other plants such as *Quercus aliena* (Di 2017) and *Parrotia subaequalis* (Zhang 2018). CpDNA, as a maternally inherited marker, primarily reflects seed dispersal and historical population formation events (Irwin 2002). The significant phylogeographic structure, high inter‐population genetic differentiation and limited gene flow detected by cpDNA indicate that seed‐mediated gene flow is highly limited in *C. axillaris* . This limited seed dispersal has led to the accumulation of genetic divergence among populations, the formation of region‐specific phylogenetic lineages and the retention of ancestral genetic signatures in glacial refugia. Whereas ITS, as a biparental inherited marker, responds to both pollen and seed‐mediated gene flow (Bradburd et al. 2013; Zhang et al. 2016). *C. axillaris* , as a tree species pollinated by both wind and insects, exhibits extensive pollen production and efficient pollen dispersal; however, its seeds are difficult to disperse over long distances, which can be explained by its biological characteristics (Chanthorn and Brockelman 2008). The non‐significant phylogeographic structure, low inter‐population differentiation and extensive gene flow revealed by ITS demonstrate that insect‐assisted pollen dispersal in *C. axillaris* is efficient and far‐reaching. This extensive pollen flow can effectively break down geographic barriers, homogenise the nuclear genetic background among populations, weaken the phylogenetic divergence caused by limited seed flow and blur the phylogeographic structure. The combination of cpDNA and ITS markers enables us to distinguish the independent contributions of historical seed‐mediated gene flow and contemporary pollen‐mediated gene flow. Specifically, cpDNA records the historical phylogenetic differentiation and isolation events of *C. axillaris* populations, while ITS reflects the extensive genetic connectivity maintained by long‐distance pollen flow. The joint effects of limited seed flow (promoting phylogenetic differentiation) and extensive pollen flow (homogenising genetic variation) ultimately shape the unique phylogeographic pattern of *C. axillaris* , and together with historical population expansion events, form the current genetic structure. Therefore, this study supports the inference that extensive pollen flow and limited seed flow jointly shape the genetic structure of *C. axillaris* .

The Mantel test results showed that neither marker exhibited a significant pattern of isolation by distance (cpDNA: *r*: −0.256, *p* = 1.000; ITS: *r*: −0.254, *p* = 1.000). This indicates that geographic isolation is not the primary factor driving the genetic differentiation of *C. axillaris* . The observed weak negative correlation likely reflects the complexity of the data structure, such as the widespread presence of main cpDNA and ITS haplotype H1 across populations due to historical expansion, rather than a biologically meaningful pattern where genetic distance diminishes with increasing geographical distance. This suggests that the currently observed genetic patterns are unlikely to have been formed by ongoing, distance‐dependent gene flow but may instead be closely related to historical population dynamics, such as fluctuations in population range (Johnson et al. 2023). On the one hand, *C. axillaris* exhibits highly efficient long‐distance pollen flow, enabling the continuous connection of geographically isolated populations and thereby inhibiting genetic differentiation based on geographic distance. On the other hand, MaxEnt modelling indicates that the species has undergone an expansion, contraction and re‐expansion pattern of distribution change since the Last Interglacial (LIG), suggesting a complex population history. These historical fluctuations may have significantly altered the original relationship between genetic distance and geographic distance. Overall, the current pattern of absence of IBD observed is most likely attributable to historical expansion events establishing a foundation of broad genetic homogeneity, which was subsequently maintained and reinforced by extensive, highly efficient pollen flow.

### 4.3 Historical Dynamics Analysis of *C. axillaris*

Neutral tests and mismatch analyses showed that *C. axillaris* had undergone significant expansion events. During the expansion process, genetic diversity rapidly accumulated, disrupting the original neutral equilibrium (Moral et al. 2009). Potentially driven by climate improvement or habitat expansion (Gosnold et al. 2011; Rubio et al. 2016). Pleistocene glacial climatic oscillations are considered major drivers of plant distribution patterns and intraspecific differentiation (Hewitt 2000, 2004). In China, six major glacial periods occurred during the Pleistocene (Cui et al. 2011). Numerous geographical studies suggest that the climatic fluctuations during the Last Glacial Maximum (LGM) and earlier cold phases significantly influenced the distribution patterns of plants in China (Qiu et al. 2011; Liu et al. 2012). Haplotype network diagrams provide insights into evolutionary relationships among haplotypes. Haplotypes located centrally within the network diagram, exhibiting higher frequency and wide geographic distribution, are generally regarded as centres of lineages differentiation (Rowe et al. 2017; Provan and Bennett 2008). In this study, the cpDNA haplotype H1 and ITS haplotype H1 both occupy central network positions and have the highest frequency as well as the widest geographic distribution, suggesting that they may be main haplotypes.

CpDNA data indicate that the GDLC population possesses the highest *H*_d_ and relatively high *π*, followed by the HNAH and HNTY populations, which harbour unique haplotypes H11 and H13/H14, respectively. GDLC population may have functioned as a gene pool during the late glacial expansion, receiving and blending genetic material from multiple refugia such as the Xuefeng Mountains‐Wushan Mountains, or the area itself may have constituted a long‐term local micro‐refuge. ITS data reveal that the HBZG population exhibits the highest *H*_d_ and *π* and possesses the unique haplotype H11. Notably, the HNAH and HNTY groups (located in the Xuefeng Mountains) are geographically adjacent to the HBZG group (located in the Wushan Mountains). These mountainous regions often serve as glacial refuges, where complex topography fosters population persistence, ecological isolation and genetic differentiation (Abebe et al. 2013). Therefore, the Xuefeng Mountain‐Wushan region is likely to have served as a potential centre of origin or important glacial refuge for *C. axillaris* .

MaxEnt modelling results further support this inference, indicating that during LIG, the region constituted a highly suitable habitat for *C. axillaris* . In contrast, most other populations outside this region contained only cpDNA or ITS haplotype H1. The LGM shows pronounced southwards contraction in suitable habitats, suggesting that *C. axillaris* likely migrated from Hunan‐Hubei southwards towards Guangdong during the LGM. Although the models predict range contractions during the LGM, our genetic data did not detect signals of a strong genetic bottleneck antecedent to the expansion. A reasonable explanation for this is that while the overall suitable habitat retreated southward during the LGM, the *C. axillaris* populations in the potential refugia we inferred (such as the Xuefeng Mountain‐Wushan region) may have maintained a sufficiently large effective population size. This would have prevented a sharp loss of genetic diversity, and thus did not produce strong signals of a genetic bottleneck. Furthermore, the decline in population size may not have reached the level of a “severe bottleneck,” or its duration may have been too short, so the genetic signal itself was weak. More importantly, the postglacial population expansion (particularly since the MH) was extremely rapid and large‐scale. This strong expansion signal likely dominates the site frequency spectrum, thereby masking or overwriting any previously existing, relatively weak signals of contraction. Additionally, the warmer and more humid climate of Guangdong may have imposed new selective pressures on these migrant populations, promoting local adaptation. This is consistent with biogeographical patterns observed in *Primula kwangtungensis* (the type specimen was collected near GDLC), which frequently occurs along the Hunan‐Guangdong border (X. Wu 2017), reflecting long‐term floristic connectivity between the two regions. This pattern of radiative dispersal from potential refugia (the Xuefeng Mountain‐Wushan region) to surrounding areas (such as Guangdong) is consistent with the classic biogeographical pattern of species expanding outward from their centre of origin (Croizat et al. 1974; Bremer 1992), indicating a complex migration history shaped by climate change.

MaxEnt predictions indicate that the current highly suitable areas for *C. axillaris* are mainly concentrated in central and southern China. This spatial distribution pattern further supports the hypothesis that multiple refugia existed during the Pleistocene glacial periods. This species exhibits high sensitivity to both annual precipitation and temperature, and its historical dispersal process can be divided into phases of expansion, contraction and re‐expansion. It is noteworthy that although annual precipitation (Bio12) was identified as the most influential variable in the model, the contribution of precipitation during the driest month (Bio14) was relatively low. This may stem from the fact that across most of the species' current distribution within the subtropical monsoon regions of Asia, even the driest months typically experience some precipitation. This implies that under contemporary climatic conditions, acute drought stress may not constitute a primary limiting factor. However, this does not prevent the possibility that Bio14 may have been a key limiting factor during extreme palaeoclimate events. During the cold and arid LGM, significantly reduced water availability likely served as the key driver for the marked southward contraction of suitable habitats for Bio14, compelling populations to retreat to refugia meeting minimum moisture thresholds. During the warm and humid LIG, its suitable habitat expanded northwards but contracted significantly southwards during the cold and dry LGM. The mid‐Holocene partially recovered, and its current distribution is relatively stable. However, under future scenarios of continued global warming, highly suitable habitats may expand northwards towards areas south of the Qinling Mountains, forming new potential habitats. Species distribution models such as MaxEnt method assume that the species is in a state of climatic equilibrium with its current environment. Given that *C. axillaris* underwent significant expansion during the post‐LGM, it may not yet have fully occupied all potential climatic niches (e.g., a state of disequilibrium), particularly along its northern expansion front. Therefore, the projected northward shift in future distribution should be understood as an indication of potential changes in climatic suitability rather than a precise prediction of migration range. Actual migration patterns will remain constrained by dispersal limitations and non‐climatic factors.

### 4.4 Conservation Implications

The findings of this study provide concrete and actionable insights for the conservation and management of *C. axillaris* :

1. Population prioritised for ex situ conservation: Priority should be given to protecting populations inferred to have served as glacial refugia, possessing high genetic diversity and unique haplotypes. Specifically, the HBZG population possesses a unique ITS haplotype (H5) and is situated within the inferred central refuge (the Xuefeng Mountain‐Wushan region), making it a key target for in situ conservation. GDLC population was identified as possessing the highest cpDNA haplotype diversity, alongside the HNAH and HNTY populations. These populations represent distinct lineages within the evolutionary history of *C. axillaris* and constitute important genetic resource pools. For populations where no variation was detected in cpDNA and ITS (such as GXSL, JXGF, JXXY, HBTS, etc.), regular genetic monitoring should be implemented. Consideration should be given to promoting gene flow with neighbouring highly diverse populations through habitat management, thereby preventing genetic drift and the loss of adaptive potential.
2. Potential corridors for assisted migration: Model projections indicate that optimal habitats will shift northwards. To facilitate potential natural dispersal of species or future assisted migration initiatives, key ecological corridors connecting current and future suitable areas should be identified and assessed. Especially, priority should be given to examining the principal river valleys (such as those of the tributaries in the middle and lower reaches of the Yangtze River) and mountain passes (such as the corridors between the Nanling Mountains and the Luoxiao and Wuyi mountain ranges) that connect the current southern highly suitable zones (e.g., the Nanling Mountains) with future northern potentially suitable zones (e.g., the Dabie Mountains). Priority should be given to safeguarding forest habitat connectivity within these corridors, thereby facilitating the natural northward migration of species. Pilot projects for assisted migration may be considered at the northern terminus of these corridors.

## 5 Conclusion

Our findings demonstrate that the current phylogeographic pattern of *C. axillaris* is the result of a combination of extensive pollen flow and recent population expansion driven by historical climate fluctuations. *C. axillaris* has undergone multiple expansions and contractions throughout its evolutionary history driven by climatic changes, particularly precipitation and temperature. During the LGM, its distribution range contracted to potential refugia such as the Xuefeng Mountains and Wushan Mountains; subsequently, during the warming period, populations expanded outward from these refugia, forming the current distribution pattern. This study not only reconstructs the dynamic processes of species' responses to past climate change but also identifies contemporary hotspots of genetic diversity and potential refugia. Concurrently, the potential northward shift of suitable habitats due to future climate warming suggests that historical migration routes and genetic adaptation potential must be fully considered when formulating ex situ conservation and assisted migration strategies.

## Author Contributions

**Peng Luo:** data curation (lead), formal analysis (lead), investigation (lead), software (lead), visualization (lead), writing – original draft (lead), writing – review and editing (lead). **Jingting Hu:** data curation (lead), formal analysis (lead), investigation (lead), software (lead), visualization (lead), writing – original draft (lead). **Mengyu Yang:** formal analysis (equal), investigation (equal), software (equal). **Xuemin Ye:** data curation (equal), investigation (equal), supervision (equal), writing – review and editing (equal). **Fei Ding:** data curation (equal), investigation (equal), supervision (equal). **Jiawei Wang:** data curation (equal), investigation (equal), supervision (equal). **Nansheng Wu:** funding acquisition (lead), project administration (lead), resources (lead), supervision (lead), writing – review and editing (lead). **Rongxi Sun:** funding acquisition (lead), project administration (lead), resources (lead), supervision (lead), writing – review and editing (lead).

## Funding

This work was supported by the National Natural Science Foundation of China, 32160387.

## Conflicts of Interest

The authors declare no conflicts of interest.

## Supporting information


**Figure S1:** Spearman analysis of 19 environmental variables.

**Figure S2:** Jackknife test result of 10 environmental variables.

**Figure S3:** AUC value obtained from ROC analysis to test model predictions.

**Figure S4:** Dominant environmental variables response curves.

**Table S1:** Primer information and Annealing temperature for *Choerospondias axillaris* .

**Table S2:** Occurrence sites from field survey and website records used for Maxent analyses in this study.

**Table S3:** Bioclimatic variables for Maxent.

**Table S4:** Polymorphic Sites of 16 Haplotypes in cpDNA Sequences of *Choerospondias axillaris* .

**Table S5:** Polymorphic Sites of 12 Haplotypes in ITS Sequences of *Choerospondias axillaris* .

**Table S6:** Contribution rate of 10 environmental variables in different periods.

**Table S7:** Predicted area of potential suitable areas under different climate scenarios of *Choerospondias axillaris* .

## Data Availability

The chloroplast DNA and ITS sequences generated in this study have been deposited in the GenBank database under accession numbers PX621079‐PX621332 (cpDNA) and PX610677‐PX610930 (ITS). All additional data, including processed data files and the results of all analyses, are provided in the Supporting Information.
