# Supplementary figures and images for: Extensive Pollen Flow and Historical Population Expansions Shaped the Phylogeographic Pattern of Choerospondias axillaris: Evidence From Chloroplast DNA and ITS Sequences
Source: Ecol Evol. 2026 Apr 30;16(5):e73588. doi: 10.1002/ece3.73588 (PMC13130045; doi:10.1002/ece3.73588)

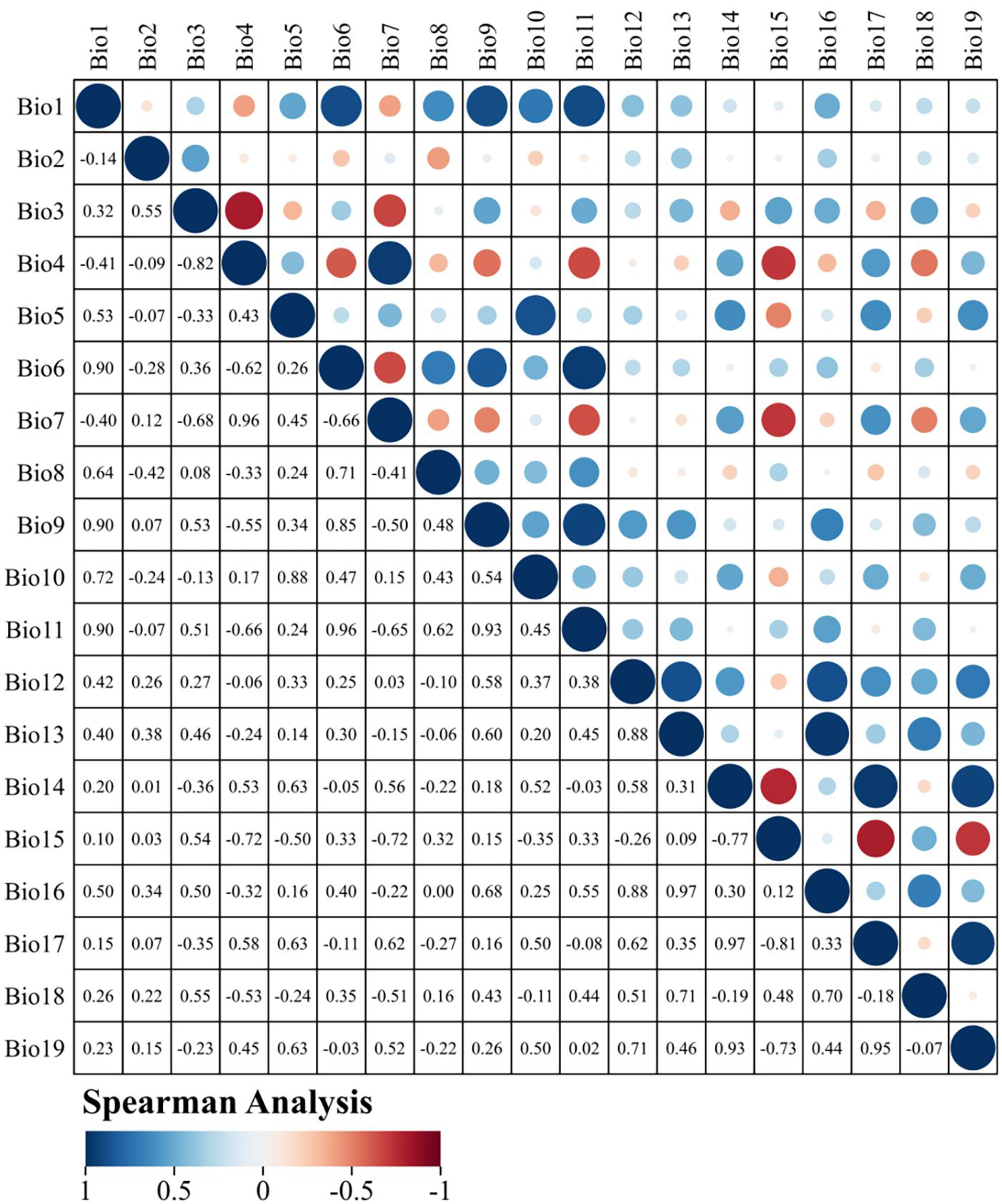

Supplement: Supplementary file 1 — Figure S1: Spearman analysis of 19 environmental variables. [file ECE3-16-e73588-s005.png]

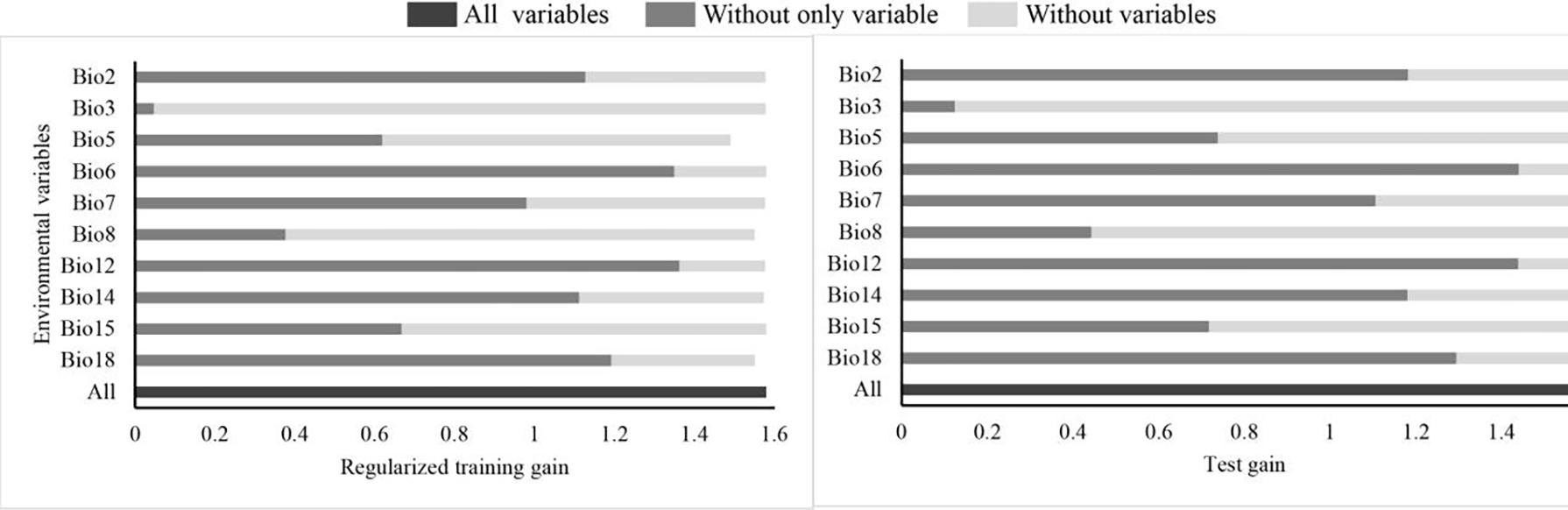

Supplement: Supplementary file 2 — Figure S2: Jackknife test result of 10 environmental variables. [file ECE3-16-e73588-s009.png]

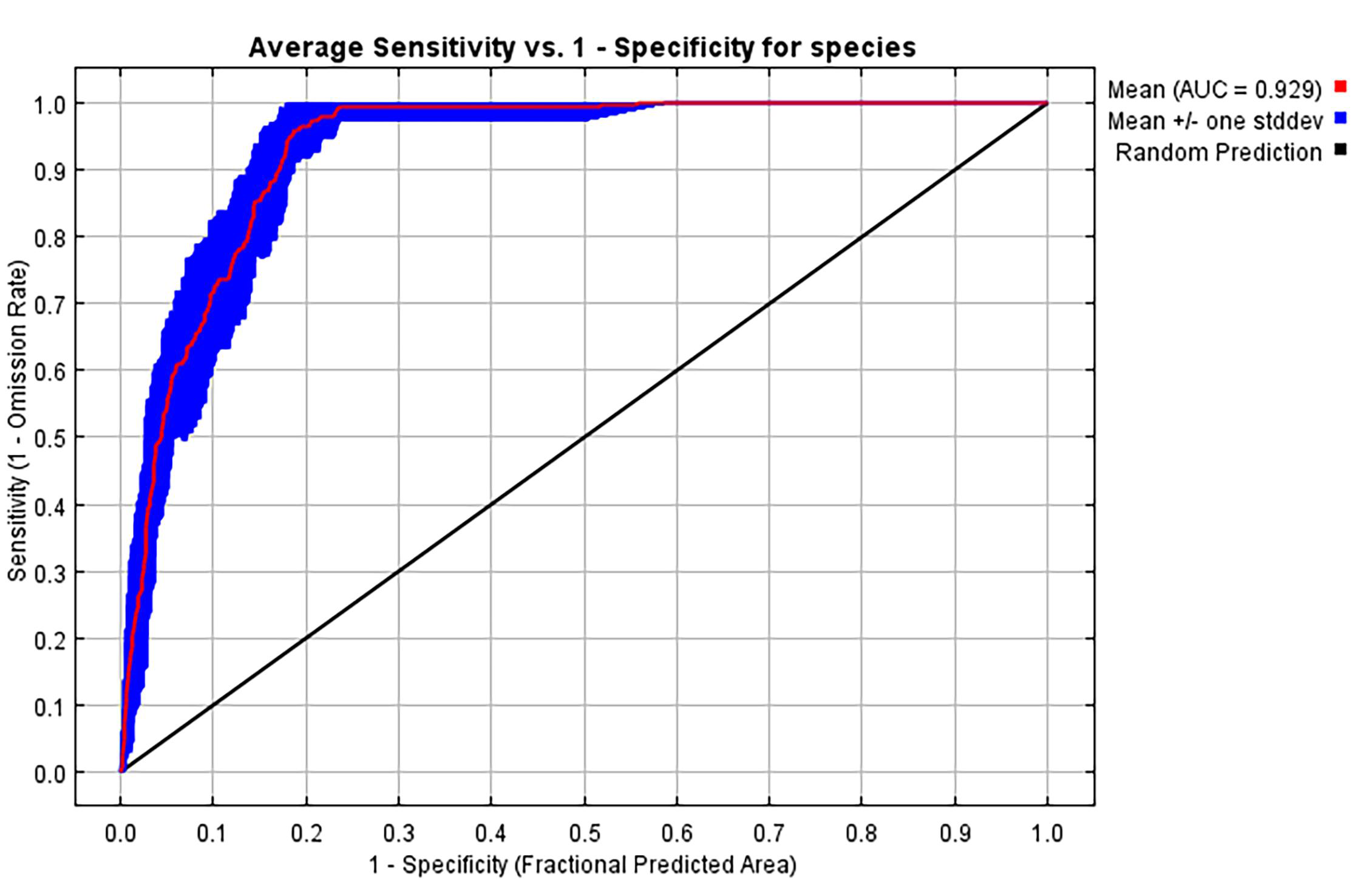

Supplement: Supplementary file 3 — Figure S3: AUC value obtained from ROC analysis to test model predictions. [file ECE3-16-e73588-s010.png]

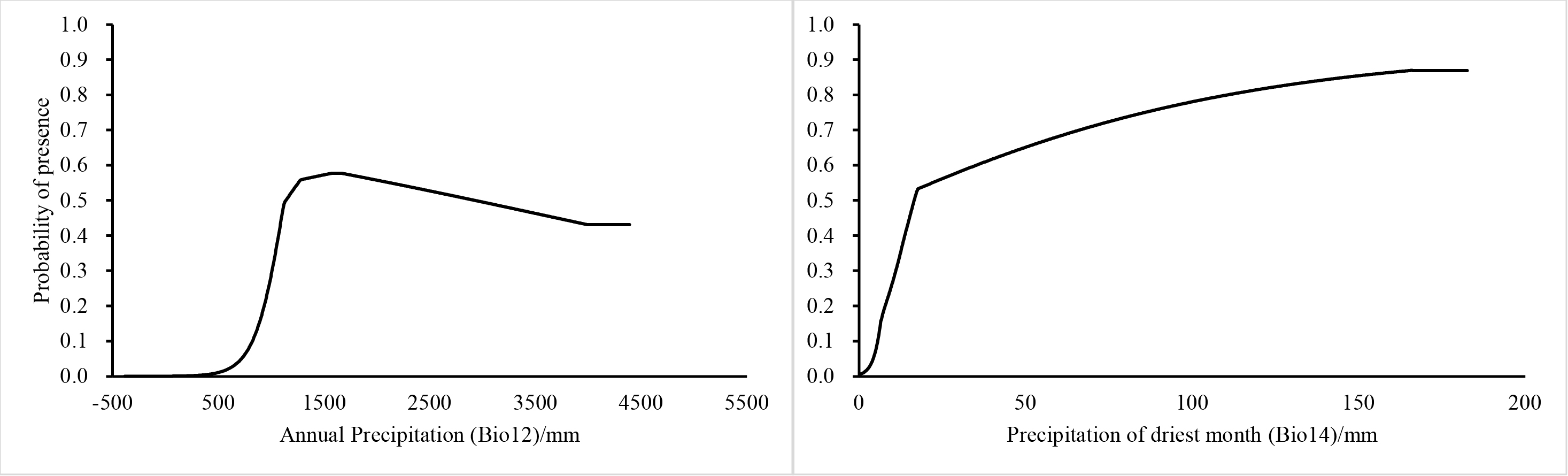

Supplement: Supplementary file 4 — Figure S4: Dominant environmental variables response curves. [file ECE3-16-e73588-s007.png]
